# Chemical fertilization: a short‐term solution for plant productivity?

**DOI:** 10.1111/1751-7915.13515

**Published:** 2019-11-27

**Authors:** Carlos Molina‐Santiago, Miguel A. Matilla

**Affiliations:** ^1^ Departamento de Microbiología, Instituto de Hortofruticultura Subtropical y Mediterránea ‘La Mayora’ Universidad de Málaga ‐ Consejo Superior de Investigaciones Científicas (IHSM‐UMA‐CSIC) Universidad de Málaga, Bulevar Louis Pasteur 31 (Campus Universitario de Teatinos) 29071 Málaga Spain; ^2^ Department of Environmental Protection Estación Experimental del Zaidín Consejo Superior de Investigaciones Científicas Prof. Albareda 1 Granada 18008 Spain

## Abstract

The effect of long‐term chemical fertilization on plant‐microorganisms and microbe‐microbe interactions.

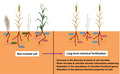

An increasing world population and climate change are global challenges that, amongst others, burden the sustainability of agricultural production. In fact, in order to feed the growing world population, current estimates indicate that our global agricultural output will need to be increased by at least 50% by 2050 (Muller *et al.*, 2017). However, the urgent demand to intensify agricultural production is challenged by diseases caused by plant pathogens along with soil erosion and land degradation. Factors affecting soil structure and degradation are multiple and include water and wind erosion, but also those derived from various human activities such as pollution or an excessive application of fertilizers. In this sense, although nutrients in soils are key for an optimal plant growth and yield, the excessive and continued application of chemical fertilizers has been shown to affect soil health by altering, for example, its microbial diversity, organic matter content and other physico‐chemical properties (Singh, 2018; Shah and Wu, 2019). For this reason, one of the main global challenges at present is to develop efficient agro‐biosystems that permit an adequate agricultural productivity with a minimal impact on the environment as well as on public health.

The interactions between plants and non‐pathogenic bacteria have been largely studied, and the advantages that both partners obtain from such association have been explored in different model systems (Berg and Koskella, 2018; Matilla and Krell, 2018; Thomashow *et al.*, 2019). In order to efficiently colonize plant roots, bacteria have developed multiple strategies. Among them, chemotaxis to plant root exudates has been shown to be important for efficient root colonization in multiple beneficial plant‐associated bacteria (Feng *et al.*, 2019; Lopez‐Farfan *et al.*, 2019). Following plant colonization, plant‐associated bacterial communities can promote plant growth and health by enhancing the uptake and availability of nutrients, synthesizing phytohormones and providing resistance against pathogens (Matilla and Krell, 2018; Biessy *et al.*, 2019; Su *et al.*, 2019). Consequently, beneficial plant‐associated bacteria are of increasing agricultural interest due to their potential as efficient alternatives to chemical products in modern agricultural practices (Matilla and Krell, 2018; Sessitsch *et al.*, 2018; Wu *et al.*, 2019). As a result, and as emphasized in a recent report published in Microbial Biotechnology, current estimates indicate that the global microbial biopesticides and biostimulants market will reach around 11 billion dollars by 2025 (Sessitsch *et al.*, 2018).

The emergence of the above‐mentioned next‐generation green revolution aimed at developing sustainable alternatives to improve crop production is also reflected in the increasing number of articles that are focused on investigating the composition of soil microbiota and how soil treatments alter bacterial soil communities. Among them, there is a remarkable number of studies that investigate the impact of long‐term fertilization, chemical or organic, on the diversity of plants and microorganisms. In general, these studies reveal that chemical fertilization reduces the diversity of plants and microorganisms as well as negatively impacts plant–microbe interactions and the soil microbiome’s capacity to contribute to soil nutrient cycling (Pierik *et al.*, 2011; Cassman *et al.*, 2016; Wang *et al.*, 2018; Li *et al.*, 2019). However, these alterations have been suggested to be also dependent on the length of the fertilization process (Pierik *et al.*, 2011; Li *et al.*, 2019), and there is a paucity of studies that investigate the effect of fertilizations lasting more than a century on the interaction between plants and microorganisms. To cast light into this aspect, an elegant multidisciplinary study by Huang *et al. *(2019) recently published in Microbial Biotechnology investigated the effect of a fertilization process lasting more than 150 years on the networks between plants and their associated functional microbial communities. Given the length of this treatment, the effect of the chemical fertilization on the soil can be considered a stable change and, consequently, this experimental approach is an excellent model to determine the impact of long‐term human‐induced alterations on soil bacteria–plant interactions. In this study, Huang *et al., *(2019) found that all major physico‐chemical properties of the fertilized soil (e.g. pH, moisture, total carbon and nitrogen) were altered as compared to the non‐treated soil. Significantly, these changes were associated with an abrupt reduction in both diversity and composition of plant species as well as in the diversity of soil microorganisms. Importantly, the use of high‐throughput approaches for functional activity analysis of microbial communities allowed the authors to determine that the number of genes involved in the degradation of recalcitrant compounds, denitrification, nitrogen fixation and phosphate utilization was largely reduced. Further *in silico* analysis revealed that long‐term fertilization not only reduced the interactions between soil microbes but also the complexity of the networks between plants and functional microbial communities. Thus, while five plant species showed an association with microbial functional genes in unfertilized soils, only one plant species showed a connection with microbial functional groups in long‐term fertilized soils. Finally, the authors showed that carbon and nitrogen contents in soil were the main parameters that modulate plant and microbe networks. They further highlighted that functional microbial communities play an important role in plant diversity (Huang *et al.*, 2019).

Anthropogenic changes in natural ecosystems are dramatically affecting global diversity. In particular, the soil microbiome plays a key role in modulating plant diversity as well as nutrient retention and recycling (Geisen *et al.*, 2019). Therefore, a reduction in the microbial biodiversity of soils may have serious consequences on the normal functioning of natural ecosystems (Geisen *et al.*, 2019). Nowadays, the intensification of agricultural production mainly depends on an increase in irrigation as well as on the use of chemical pesticides and fertilizers (Sessitsch *et al.*, 2018; Geisen *et al.*, 2019). These practices not only result in an increased emission of greenhouse gases or in a decreased water availability, but also substantially affect the biodiversity of soil microorganisms and plants, as shown by Huang *et al. *(2019). Consequently, future strategies should explore the sustainability of our agricultural systems. Among the options to be considered, soil microbiome management represents a promising approach to maintain the well‐balanced soil microbial diversity that is essential for plant health and crop productivity.

## Conflict of interest

None declared.
